# Circulating miRNA Expression Is Inversely Correlated with Tumor Tissue or Sentinel Lymph Nodes in Estrogen Receptor-Positive Early Breast Cancer Patients

**DOI:** 10.3390/ijms241713293

**Published:** 2023-08-27

**Authors:** Daniel Escuin, Laura López-Vilaró, Olga Bell, Josefina Mora, Bárbara García-Valdecasas, Antonio Moral, Montserrat Clos, Laia Boronat, Cristina Arqueros, Agustí Barnadas

**Affiliations:** 1 Institut d’Investigació Biomèdica Sant Pau (IIB-Sant Pau), 08041 Barcelona, Spain; llopezv@santpau.cat (L.L.-V.); obell@santpau.cat (O.B.); 2 Department of Pathology, Hospital de la Santa Creu i Sant Pau, 08041 Barcelona, Spain; 3 Department of Biochemistry, Hospital de la Santa Creu i Sant Pau, 08041 Barcelona, Spain; jmora@santpau.cat; 4 Department of Surgery, Hospital de la Santa Creu i Sant Pau, 08041 Barcelona, Spain; bgvaldecasas@santpau.cat (B.G.-V.); amoral@santpau.cat (A.M.); mclos@santpau.cat (M.C.); 5 Faculty of Medicine, Universitat Autònoma de Barcelona (UAB), 08193 Bellaterra, Spain; 6 Department of Medical Oncology, Hospital de la Santa Creu i Sant Pau, 08041 Barcelona, Spain; lboronat@santpau.cat (L.B.); carqueros@santpau.cat (C.A.); 7 Centro de Investigación Biomédica en Red Cáncer (CIBERONC), 28029 Madrid, Spain

**Keywords:** miRNAs, early breast cancer, sentinel lymph node, metastasis, biomarkers

## Abstract

The deregulation of microRNAs (miRNAs) is associated with the various steps of the metastatic process. In addition, circulating miRNAs are remarkably stable in peripheral blood, making them ideal noninvasive biomarkers for disease diagnosis. Here, we performed a proof-of-principle study to determine whether tumor-tissue-derived miRNAs are traceable to plasma in ER-positive early breast cancer patients. We performed RNA-sequencing on 30 patients for whom plasma, sentinel lymph nodes (SLNs) and tumor tissue were available. We carried out differential expression, gene ontology and enrichment analyses. Our results show that circulating miRNAs are inversely expressed compared with tumor tissue or SLNs obtained from the same patients. Our differential expression analysis shows the overall downregulation of circulating miRNAs. However, the expression of miR-643a-3p and miR-223 was up-regulated in patients with positive SLNs. Furthermore, gene ontology analysis showed the significant enrichment of biological processes associated with the regulation of epithelial cell proliferation and transcriptional regulation commonly involved in the promotion of metastases. Our results suggest the potential role of several circulating miRNAs as surrogate markers of lymph node metastases in early breast cancer patients. Further preclinical and clinical studies are required to understand the biological significance of the most significant miRNAs and to validate our results in a larger cohort of patients.

## 1. Introduction

Cancer metastases are responsible for the majority of breast cancer deaths. Despite intensive research in this field, our understanding of the molecular events that drive metastatic progression remains largely incomplete. In recent years, microRNAs (miRNAs) have emerged as important regulators of many cellular processes, including the various steps of the metastatic process. miRNAs are small (19–25 nt), noncoding RNA (ncRNA) molecules that regulate the expression of target genes by binding to complementary regions of mRNAs to repress their translation or regulate their degradation. Many cellular pathways are affected by the regulatory function of miRNAs, and several human pathologies, including cancers, are associated with the deregulation of miRNAs [1] and their metastases [2].

The development of miRNA signatures in liquid biopsies for the diagnosis of a variety of different cancer types is an active field of research [3]. This is in part due to the fact that circulating miRNAs and other small RNA molecules are stable in peripheral blood [4,5], making them ideal noninvasive biomarkers for disease diagnosis. Our recent research has focused on the involvement of miRNAs in the development of locoregional metastases in patients with early breast cancer [6]. We showed a good correlation in the miRNA expression profile between sentinel lymph nodes (SLNs) and matched primary tumor samples, in agreement with a previous study [7]. The down-regulation of various miRNAs was significantly differentially expressed in patients with locoregional metastasis. In addition, we recently profiled the expression of miRNAs in a small number of plasma samples collected before surgery from patients with early-stage breast cancer who were not previously treated [8]. Our results showed an overall downregulation of miRNAs that, in many cases, are direct targets of proteins that promote metastasis. One important caveat in our previous studies was a lack of correlation between miRNA expression in plasma, tumor tissues and SLNs obtained from the same patients. This is of utmost importance in order to develop potential circulating-based biomarkers. First, to elucidate whether circulating miRNAs are derived from tumor-tissue miRNAs and, second, to establish whether plasma miRNAs accurately reflect the heterogeneity of a primary tumor and its subclones [3].

Here, we performed RNA-sequencing to profile the expression of miRNAs in 30 early breast cancer patients for whom plasma, tumor tissue and SLN samples were available. Our results show an inverse relationship between circulating miRNAs and tumor/SLN samples. Nonetheless, several circulating miRNAs were associated with metastatic SLN status via a significant enrichment of the biological processes associated with the regulation of epithelial–mesenchymal transition, cell proliferation and transcriptional regulation. Our data highlight the potential use of circulating miRNAs as surrogate markers of locoregional metastases in breast cancer.

## 2. Results

### 2.1. Patients

A total of 30 female patients were included in this study. We analyzed 30 plasma samples, 29 SLNs (2 SLNs were collected from the same patients) and nine tumor tissues. We paired plasma and SLN samples for 27 patients; paired plasma and tumor tissue for 9 patients; and all three samples available for 7 patients (Table S1). The main clinicopathological characteristics of the patients are described in Table 1. A total of 12 (40%) patients had SLN-positive tumors; seven SLNs were diagnosed as micrometastasis (23%); and five were diagnosed as macrometastasis (17%).

### 2.2. RNA-Sequencing

All samples passed pre- and post-sequencing quality checks, which confirmed average read quality and base quality Q-scores of >30 (99.9% correct) [9] and the expected read length distribution for miRNAs (Figure S1). The mean read numbers for plasma and tissues were 18.5 ± 0.9 million and 13.8 ± 0.6 million, respectively. Following sequencing and trimming, all reads containing identical insert sequences and UMI sequences (insert-UMI pair) were collapsed into a single read and passed into the analysis pipeline. This allowed for the true quantification of the miRNAs by eliminating library amplification bias and a better representation of the RNA molecules in the sample. We obtained an average of 1.97 million and 2.24 million collapsed reads for plasma and tissues, respectively, which resulted in good miRNA mapping reads with a very dominant miRNA peak in all samples, indicating good sample/data quality (Figure S2A). Overall, we obtained average genome mapping rates of 65% and 95% for plasma and tissues, respectively (Figure S2B). After mapping and counting relevant entries in mirbase_22, the expression of known miRNAs was calculated using the TPM method (Table S2). We did not identify any sequences identical to those of known miRNAs in miRBase_22 for other organisms.

### 2.3. Unsupervised Clustering Analysis

**Figure 1:**
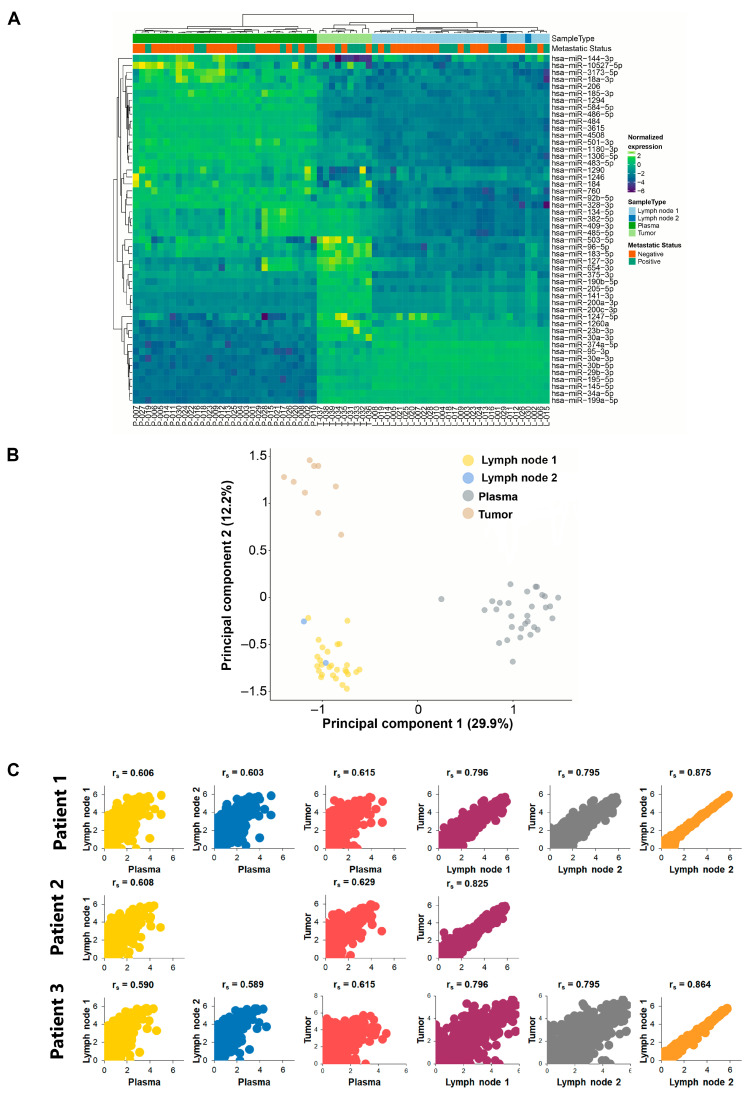
**Class discovery associated with SLN metastatic status.** The analysis was performed using the 50 miRNAs with the largest coefficients of variation based on the trimmed mean of M-values (TMM counts). (**A**) Heat map and unsupervised hierarchical clustering. Each row represents one miRNA, and each column represents one sample. The color represents the relative expression level of a miRNA across all samples. The color scale shows the expression level above (purple) or below (green) the mean. (**B**) Principal component analysis (PCA) shows sample clusters arising naturally based on the miRNA expression profile. Tumors are shown in light brown, plasma samples in grey and SLNs in yellow. Patients with more than 1 SLN analyzed are depicted as blue. (**C**) Representative scatterplots of selected patients showing a sample-to-sample comparison between different tissues from the same patient as indicated. The scatterplots show the log expression of miRNA expression and the Spearman correlation coefficient (r_s_) for each comparison.

We investigated whether patients were assigned to biological groups based on their miRNA expression. We performed a supervised two-way hierarchical clustering of miRNAs and samples using the 50 miRNAs with the largest coefficients of variation based on TMM counts. Interestingly, the expression of circulating miRNAs showed an inverse correlation with either tumor tissues or SLNs obtained from the same patients. That is, for all patients studied, we observed that any miRNAs overexpressed in tissues were downregulated in plasma and vice versa (Figure 1A and Figure S3). We obtained similar results using a principal component analysis (PCA), which clearly clustered plasma samples into a different group (Figure 1B). In addition, we performed Spearman’s correlation analysis (r_s_) between samples obtained from each patient. Our data show that both tumor and SLN samples from the same patient have a high correlation index, with an average value of r_s_ = 0.78 among all patients. Interestingly, we obtained two SLNs from patients 1 and 20. The latter patient had both SLNs diagnosed as macrometastases, whereas the former patient had one macrometastasis and one micrometastasis. In both cases, we showed the highest correlations with r_s_ = 0.875 and r_s_ = 0.864, respectively (Figure 1C). In contrast, both tumors and SLNs showed little correlation when compared with plasma samples from the same patient, with averages of r_s_ = 0.61 and r_s_ = 0.59, respectively (Figure 1C and Figure S4).

### 2.4. Differentially Expressed Circulating miRNAs

**Figure 2:**
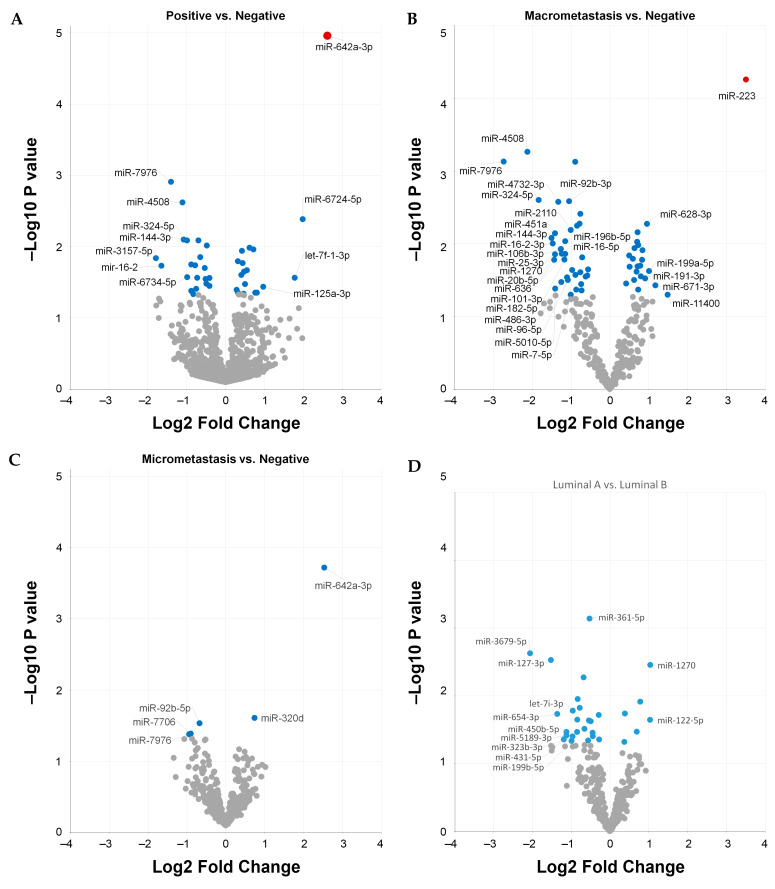
**Differentially expressed circulating miRNAs**. The volcano plots show differentially expressed miRNAs in plasma samples according to the patients’ locoregional metastatic status as indicated (**A**–**C**) or the molecular subtype (**D**). The data show the logarithmic relationship between non-adjusted *p*-values (*y*-axis) and the fold change expression (*x*-axis). Corrected q-values of <0.05 are shown as red dots; non-adjusted *p*-values of <0.05 are shown as blue dots; and non-significant *p*-values of >0.05 are shown as gray dots. Only miRNAs with an absolute log2 fold change of ≥1 are labeled.

We focused on circulating miRNAs to investigate whether differentially expressed plasma miRNAs were associated with the locoregional metastasis status of the patients. First, we analyzed plasma samples according to positive (*n* = 12) or negative (*n* = 18) SLN metastasis status. We found 34 miRNAs with a significant differential expression (*p* < 0.05). However, only the upregulation of hsa-miR-642a-3p remained significant after correcting for multiple testing (q = 0.034) (Figure 2A). Overall, we found four miRNAs upregulated and seven miRNAs downregulated with an absolute log2 fold change of ≥1 (Table S3). Similar results were found when patients were sub-classified into positive macrometastasis (*n* = 5) or micrometastasis (*n* = 8) SLNs and compared with patients with negative SLNs (*n* = 18). We found that hsa-miR-642a-3p remained upregulated in patients with micrometastasis SLNs (*p* < 0.05) but not in patients with macrometastasis SLNs. In contrast, we found that hsa-miR-223 was upregulated (q = 0.03) in patients with positive macrometastasis (Figure 2B). Since our series included patients with luminal A and luminal B tumors (Table 1), we analyzed the differential expression between these two subgroups. Our results show 12 miRNAs with an absolute log2 fold change of ≥1 when comparing luminal B against luminal A tumors. Ten miRNAs were downregulated and two miRNAs were upregulated in luminal B tumors. However, none of them remained significant after correcting the *p*-values for multiple testing (q > 0.05) (Figure 2D, Table S3).

### 2.5. Biological Significance and Enriched Analysis of Circulating miRNAs

**Figure 3:**
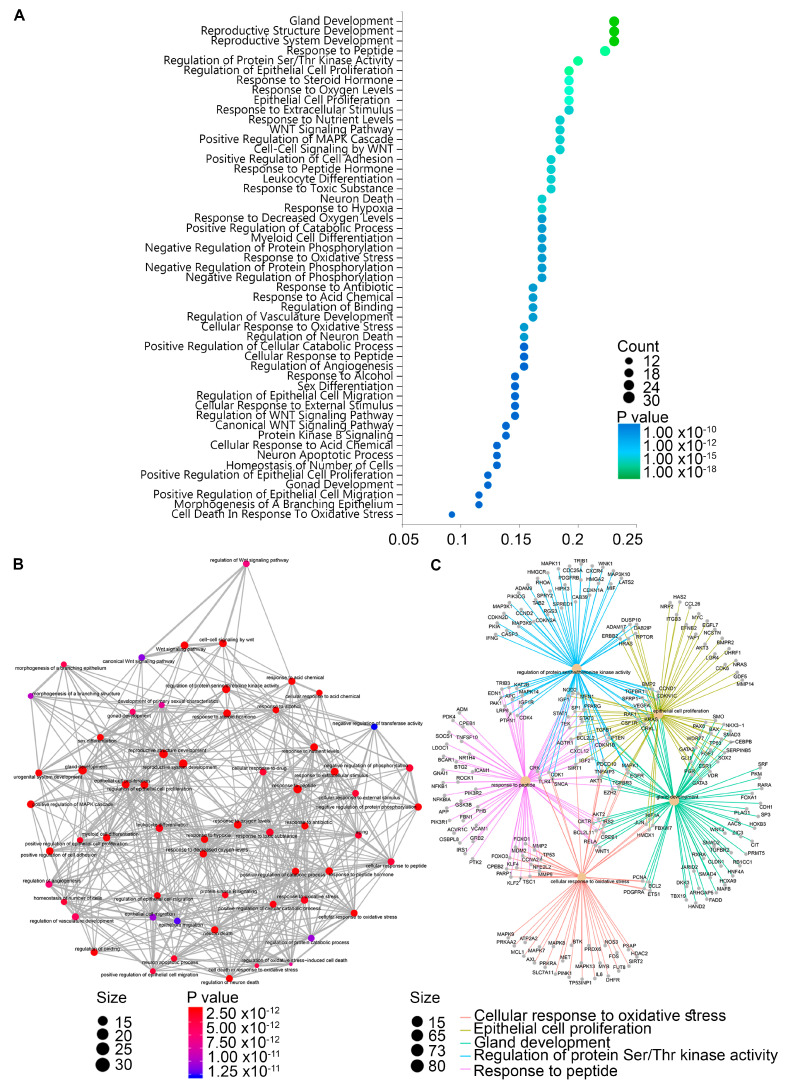
**Gene ontology (GO) enrichment analysis for significant biological processes associated with positive SLNs.** (**A**) Dot plot graph shows the 50 most significant biological-process GO terms (*y*-axis) and the ratio between the number of expressed miRNAs associated with the GO term and the number of significantly differentially expressed genes associated with the GO term (*x*-axis). The colors of the nodes indicate the *p*-value and the size of the nodes denotes the number of miRNAs associated with a specific GO term. (**B**) Enrichment map of the top 60 miRNAs. Pathways are grouped by similarity. The size of each node indicates the number of miRNAs found in a pathway, whereas the color of the node is based on the significance of the *p*-value (**C**) Neural network shows the GO terms for the biological processes associated with patients with positive SLNs.

We performed a biological significance analysis using a subset of miRNAs (*p* < 0.05 and absolute log fold change >0.3). Table 2 shows a summary of the top targeted genes for each miRNA investigated. A complete list of validated target genes and all evidence types are provided in Table S4. In addition, we carried out an enrichment analysis of the GO and Reactome Pathways databases using the identified set of *Homo sapiens* target genes to understand how our data are related to biological functions. The results are summarized in Figure 3 and Table 3. Our data show that differentially expressed miRNAs were associated with biological processes (BP) markedly focused on gland development, gene expression regulation via receptor tyrosine kinases, epithelial cell motility and proliferation processes (*p* < 0.001). A complete list of the analyses for each comparison is included in Table S5. Differentially expressed miRNAs were enriched in cellular component (CC) terms located in membrane fractions, WNT signalosome and transcription factor complexes. Moreover, differentially expressed miRNAs were enriched in molecular function (MF) terms associated with RNA polymerase II, chromatin and ubiquitin-binding (*p* < 0.001) (Table 3).

### 2.6. Clinicopathological Correlation with Circulating miRNAs

Our series included 30 patients with ER+ early breast cancer, and we reported recurrence in two (7%) patients. The median follow-up time was 5.2 years (range 2.3–7.3 years). At the last follow-up, all were reported to be alive. We investigated whether the differentially expressed miRNAs correlated with the patients’ clinicopathological parameters. The expressions of miR-6724-5p, miR-27a-3p, miR-423-3p and miR-140-5p were significantly higher in patients with high Ki67. The expression of miR-16-2 was significantly higher in older patients (>60 years). The expressions of miR-6724-5p, miR-320d and miR-423-3p were significantly higher in luminal B tumors. The increased expression of miR-182-5p was associated with higher-grade tumors, and low miR-3157-5p expression was associated with the presence of tumor multifocality. Given the low number of events, we were unable to perform any survival analysis on our cohort of patients.

## 3. Discussion

In recent years, miRNAs have emerged as important regulators of the various steps of the metastatic process [10]. Currently, lymph node affection remains the most important prognosis factor in breast cancer [11], and the presence of metastasis in SLNs is still the recommended procedure for the axillary staging of early breast cancer. Our recent research focused on the involvement of miRNAs in the development of locoregional metastases in patients with early-stage breast cancer. Our initial study focused on profiling the expression of miRNAs via RNA-seq in paired tumor tissue and lymph nodes from the same patients [6], whereas the second was a small observational study of plasma samples in breast cancer patients [8]. Both studies reported an association between several miRNAs and the locoregional metastatic outcomes of patients. However, we could not establish whether the expression of circulating miRNAs resembled that of primary tumors or SLNs since both sample cohorts were obtained from different patients.

Therefore, in this study, we sought to address this issue by analyzing plasma, SLNs and tumor tissues derived from the same patients and correlating the data with the metastatic status of these patients. In total, we RNA-sequenced 30 plasmas, 30 SLNs and nine tumor tissues (*n* = 68 samples) from 30 patients with ER+ early breast cancer. Overall, our data show that the miRNA expression in tumors and SLNs is similar, with minor changes that are likely due to histological differences between the SLN and the tumor. In contrast, the expression of circulating miRNAs showed an inverse correlation with tumor and SLN samples. For instance, any specific miRNAs that were upregulated in tumor tissues or SLNs were downregulated in plasma samples and vice versa. In fact, when we performed a sample-to-sample comparison on the same patients, the plasma samples showed a low Spearman correlation when compared with both tumor tissues or SLNs. Our data agree with previous publications, suggesting little correlation between the tumor and serum expression of miRNAs in breast cancer using qPCR [12]. However, we cannot rule out the possibility that blood sample collection may influence the expression of circulating miRNAs. In this regard, there is evidence showing that, in metastatic colorectal cancer, circulating miRNA expression changes before and after passing through the metastatic site (liver), as if somehow some miRNAs are retained specifically in the liver, while others are not [13]. In addition, transcriptome analysis revealed non-identical miRNA profiles between arterial and venous plasma [14]. Similarly, comparisons between arterial and venous miRNAs in hypertension-versus-control conditions also revealed a prominent disease association between circulating miRNAs and their target genes in arteries but not in veins [15]. The overall picture that emerges from these studies is that the blood collection point affects the miRNA expression profile. Increasing evidence also shows that tumor-draining veins constitute a better source of biomarkers than peripheral veins in colon cancer patients [16]. For instance, a recent small exploratory study indicated that tumor-proximal venous samples are highly enriched in miRNAs, ctDNA mutations and circulating tumor cells among oncological biomarkers and may allow for more robust molecular analysis than peripheral vein samples [17,18]. Whether something similar occurs in breast cancer patients remains to be answered. In our study, all plasma samples were obtained from a peripheral vein, and this could, at least in part, explain the observed discrepancies between circulating miRNAs and paired tumors or SLNs.

Despite all the aforementioned data, we identified several circulating miRNAs associated with the locoregional metastatic statuses of patients. Overall, we found that most differentially expressed miRNAs associated with positive SLNs were downregulated (*p* < 0.05), in agreement with our previous study, including eight miRNAs (339-5p, miR-29b-3p, 139-5p, 144-3p, 423-3p,584-5p, 99b-3p and 101-3p) that we previously described [8]. However, their differential expression did not remain significant after adjusting for a false discovery rate. Interestingly, our results show the upregulation of miR-642a-3p and miR-223 (q < 0.05) in patients with positive locoregional metastases. The upregulation of miR-642a-3p has been reported to mediate the HDAC inhibitor-mediated downregulation of HER2 and apoptosis in breast cancer cell lines [19]. Others have associated the upregulation of miR-642a-3p as an invasion/metastasis associated miRNA via the mTOR and PI3K-AKT signaling pathways in gallbladder cancer [20], whereas, in breast cancer, miR-642a-3p expression increases in the breast milk extracellular vesicles of mothers with obesity and is associated with cancer signaling pathways [21]. In the case of miR-223, its role in human cancer is controversial, and it has been found recently to act as both a tumor-suppressor gene and an oncomiR [22]. This dual role may mainly depend on its target in different locations of tissues and cells, and it has been described in breast [23], pancreatic [24] and prostate [25] human cancers. Our data show an upregulation of miR-223 in early-breast cancer patients with positive macrometasis in the SLNs and, thus, support its role as an oncomiR. Interestingly, our enrichment analysis showed that miR-233 top target genes (i.e., FOXO3, MEF2C and FBXW7) have already been described as mediating miR-233 oncomiR characteristics. For instance, FoxO3a, a well-defined tumor suppressor gene in the forkhead transcription factor O subfamily (FoxO), has been shown to be downregulated by the increased expression of miR-233 in pancreatic cancer cell lines [24]. Finally, our GO analysis shows that both miR-642a-3p and miR-223 are involved in GO biological processes related to gland and reproductive development, the regulation of receptor tyrosine kinases and epithelial cell proliferation among the top significant terms, adding further evidence of their role in promoting tumor metastasis.

To conclude, in this study, we provided evidence that circulating miRNAs have an inverse correlation with the expression observed in tumor and SLN samples derived from the same patients. Therefore, we could not fully establish whether the expression of circulating miRNAs resembles that of the primary tumor or the SLNs. This is of utmost importance in developing potential circulating-based biomarkers because (1) the origin of tissue-derived miRNAs traceable in plasma and (2) to what extent circulating miRNAs accurately reflect the heterogeneity of a tumor and its subclones (or all tumor lesions in metastatic patients) need to be established [3]. Nonetheless, we described several miRNAs associated with the locoregional metastatic status of patients with breast cancer, which have been reported to be direct targets of proteins that promote metastasis. Further studies are required with a larger number of patients to demonstrate whether the sensitivity and specificity of plasma miRNAs may be improved through selective venous sampling in breast cancer patients and to unveil the molecular mechanisms of the miRNAs described in this study.

## 4. Materials and Methods

### 4.1. Patients

We studied 30 patients with early breast cancer treated with surgery. Male patients were excluded from this study. None of the patients had prior treatment with surgery, chemotherapy or radiation. All patients had confirmed diagnoses based on the histopathology of tumor biopsies and intraoperative SLNs evaluated using the OSNA assay [26]. All tumors were invasive ductal carcinomas (IDCs) with or without in situ components. All patients were estrogen receptor-positive and HER2-negative. We collected the following clinical and pathological parameters: age, menopausal status, personal and familiar disease precedents, clinical follow-up, tumor stage determined according to the UICC system [27], histological grade determined using the Elston–Ellis grading system [28], tumor histology, presence of associated carcinoma in situ, presence of vascular and lymphatic invasion, tumor-infiltrating lymphocytes, tumor focality, tumor necrosis, proliferation of non-tumoral tissue.

### 4.2. Sample Processing

All samples were collected from early breast cancer patients who had not received any previous treatment. Peripheral blood was withdrawn before surgery. Approximately 10–15 mL of peripheral blood was collected for plasma processing in EDTA tubes. Plasma tubes were processed within 2 h of collection, spun at 1200× *g* for 10 min and inspected for the absence of hemolysis, as previously described [8,29]. Tumors and SLNs were processed as previously described [6]. All samples were aliquoted in 1.5 mL tubes and stored at −80 °C until further processing.

### 4.3. RNA Isolation

RNA was isolated from 300 μL of plasma samples using the miRNeasy serum/plasma advanced kit (Qiagen, Germantown, MD, USA) according to the manufacturer’s instructions. RNA was eluted in a volume of 14 μL. RNA was isolated from tumor tissues and SLNs using the miRNeasy kit (Qiagen, Germantown, MD, USA) according to the manufacturer’s instructions and eluted in a volume of 30 μL. The RNA integrity (RIN) level was measured for each RNA sample using the Agilent TapeStation (Santa Clara, CA, USA). All samples used in this study had a RIN value > 7. A range of spike-ins was added to all samples prior to RNA isolation. A pre-sequencing quality check using q-PCR was performed on all samples to control for the quality of the RNA and the inhibition of enzymatic reactions in downstream reactions, as previously described [8].

### 4.4. RNA-Sequencing

Next-generation sequencing (NGS) and genome annotation were performed as previously described [6,8]. Genome annotation was performed using the QIAGEN CLC Genomics Server v20.0.4 (Qiagen, Germantown, MD, USA). Following sequencing, Cutadapt (1.9.1) [30] was used to trim adaptor sequences. A quality check (QC) was performed to ensure Q-scores > 30 (>99.9% correct) for our data [9]. Reads with the correct length were analyzed for the presence of UMIs using Cutadapt v1.9.1 (Dortmund, Germany) and then collapsed by UMIs into FASTQ files. This approach eliminated library amplification bias and allowed for the true identification of miRNAs. The Bowtie2 software v2.2.6 (San Diego, CA, USA) was used to map the reads. The mapping criterion for aligning reads to spike-ins, abundant sequences and miRBase_22 was for reads to have perfect matches with the reference sequences. To map the genome, one mismatch was allowed in the first 32 bases of the read. Small insertions and deletions (INDELs) were not allowed. The resulting sequences were annotated using human assembly GRCh38 (Ensembl) and the miRBase_22 database. Transcripts per million (TPM) was used as a normalization procedure to correct for differences in sequencing depth and to quantify each RNA species.

### 4.5. Differential Expression Analysis

Differential expression analyses were performed using the TMM normalization method [31] and the EdgeR v3.17 statistical software package (Bioconductor; Victoria, Australia). We used TMM-normalized quantification R scripts [32] to perform principal component analysis (PCA) and unsupervised hierarchical clustering analysis. We selected the top 50 miRNAs with the largest coefficient of variation across all samples to obtain a cluster of samples. The data were normalized to TMM and converted to log2 scale.

### 4.6. Biological Significance Analysis, Gene Ontology (GO) and Enrichment Analysis

A search of validated gene targets was performed with multiMiR Bioconductor’s package (http://multimir.ucdenver.edu last accessed on 7 July 2023) [33], which enables the retrieval of miRNA–target interactions from different databases like mirecords, mirtarbase and tarbase. Only miRNA–target interactions with strong experimental evidence (i.e., luciferase assay or Western blot) were considered. Gene ontology (GO) [34] and Reactome Pathway [35] analyses were performed with R/Bioconductor’s Cluster Profiler package, v3.12.0 [36], using the experimentally verified targets of significantly differentially expressed miRNAs as inputs, as previously described [6,8].

### 4.7. Statistics

Differentially expressed miRNAs from RNA-sequencing data were detected using an exact test based on conditional maximum likelihood (CML), included in the R/Bioconductor package EdgeR (Victoria, Australia) [37]. *p*-values from RNA-sequencing were corrected (q-values) for multiple testing using the Benjamini–Hochberg procedure [38]. A false discovery rate (FDR) of q < 0.05 was considered significant. In all group comparisons, missing expression values were treated as zero. Differences in total numbers of miRNAs between groups were analyzed using two-sided parametric *t*-tests. A clinicopathological analysis was performed using Student’s *t*-test for the comparison of quantitative variables and the X2 or Fisher exact tests for the comparison of qualitative variables. The Cox regression model was used to perform the multivariate analysis. A two-sided *p*-value ≤ 0.05 was considered significant.

## Figures and Tables

**Table 1:** Basic patient and tumor characteristics.

Variable		N (%)
Patients		30 (100)
Age, years	Mean + SD	63.9 ± 7.6
	Median (range)	62.4 (52.6–83.8)
Tumor stage	IA	16 (53.3)
	IIA	9 (30)
	IIB	5 (16.7)
Tumor grade	I	10 (33.3)
	II	20 (66.7)
Node status	Negative	18 (60)
	Positive	12 (40)
	Micrometastasis	7 (23.3)
	Macrometastasis	5 (16.7)
Tumor focality	Unifocal	18 (60)
	Multifocal	10 (33.3)
	Multicentric	2 (6.7)
Estrogen receptor	ER+ PR+	27 (90)
	ER+ PR−	3 (10)
Molecular subtype	Luminal A	20 (66.7)
	Luminal B	10 (33.3)
Ki67	<14%	21 (70)
	≥14%	9 (30)
Surgery	Mastectomy	8 (26.7)
	Lumpectomy	22 (73.3)
Lymphovascular invasion	Negative	29 (96.7)
	Positive	1 (3.3)

**Table 2:** **Biological significance analysis.** We analyzed a subset of differentially expressed miRNAs, and we show the top 5 validated targets for each miRNA.

Mature miRNA	Target Genes	Targets	Log FC	*p*-Value
hsa-miR-192-5p	*DHFR, WNK1, RB1, ACVR2B, AKT1*	41	−1.439	0.001
hsa-miR-376c-3p	*BCL2, BMI1, RUNX2, NR5A2, GRB2*	11	−2.432	0.001
hsa-miR-192-5p	*DHFR, WNK1, RB1, ACVR2B, AKT1*	41	−1.439	0.001
hsa-miR-193b-5p	*STMN1*	1	−1.572	0.003
hsa-miR-99a-5p	*FGFR3, AKT1, CAPNS1, FKBP5, MTOR*	15	−0.967	0.005
hsa-miR-885-5p	*CASP3, CDK2, CTNNB1, IGF1R, MCM5*	5	−1.910	0.008
hsa-miR-9-5p	*NTRK3, PRDM1, REST, FOXO1, NFKB1*	82	1.456	0.009
hsa-miR-15b-3p	*CCND3, IGF1R, RECK, TRIM31, SIRT4*	5	−0.647	0.010
hsa-miR-92b-3p	*CDKN1C, ITGA6, ITGAV, SMAD3, SMAD7*	13	−0.624	0.010
hsa-miR-425-5p	*CCND1, BTK, FGFR3, SMAD2, PTEN*	9	−0.484	0.010
hsa-let-7a-5p	*NF2, TRIM71, HMGA2, CASP3, DICER1*	55	−0.437	0.011
hsa-miR-320d	*GNAI1, RBFOX2*	2	0.616	0.013
hsa-miR-194-5p	*ACVR2B, BMI1, BMP1, CDH2, CDKN1B*	23	−0.826	0.013
hsa-miR-486-5p	*ARHGAP5, CDK4, DOCK3, FBN1, FOXO1*	14	−0.491	0.015
hsa-miR-629-5p	*HNF4A, TRIM33*	2	0.471	0.015
hsa-miR-125b-5p	*ERBB2, ERBB3, BAK1, NTRK3, SMO*	122	0.599	0.018
hsa-miR-92a-3p	*ITGA5, BMPR2, CD69, CDH1, DNMT1*	38	−0.498	0.018
hsa-miR-27a-3p	*SP1, SP3, SP4, RUNX1, FOXO1*	67	0.362	0.018
hsa-miR-223-3p	*FOXO3, FOXO1, FBXW7, IGFR1, MEF2C*	67	0.812	0.018
hsa-miR-625-3p	*MAP2K6*	1	−0.623	0.019
hsa-miR-96-5p	*FOXO1, IRS1, AQP5, CELSR2, ODF2*	31	−0.848	0.021
hsa-miR-122-5p	*CCNG1, BCL2L2, ADAM17, NDRG3, ADAM10*	67	−1.286	0.021
hsa-miR-378a-3p	*RUNX1, CDK6, CYP19A1, GLI3, GRB2*	19	−0.514	0.022
hsa-miR-25-3p	*FBXW7, ATP2A2, CDH1, CDKN1C, CYP2B6*	29	−0.648	0.023
hsa-miR-483-3p	*CDK4, CCN2, IGF1, SMAD4, RASGRF1*	10	−1.207	0.023
hsa-miR-642a-3p	*GRK2, CACNA1B, CDC25B, CDKN1A, FOXN3*	82	0.796	0.023
hsa-miR-34a-5p	*E2F3, VEGFA, BCL2, MYCN, SIRT1*	127	−0.980	0.024
hsa-miR-7-5p	*IRS2, IRS1, EGFR, RAF1, PAK1*	46	−0.737	0.031
hsa-let-7b-5p	*HMGA2, CDK6, CDC25A, ACTG1, ACVR1*	40	−0.402	0.031
hsa-miR-30c-5p	*UBE2I, BCL9, CAMK2D, CASP3, RUNX2*	39	−0.475	0.033
hsa-miR-885-3p	*BMPR1A, CD274*	2	−1.663	0.034
hsa-miR-24-3p	*CDKN2A, ACVR1B, DHFR, MYC, E2F2*	84	0.301	0.034
hsa-miR-365a-3p	*HDAC4, ACVR1, BAX, CCND1, BCL2*	10	−1.078	0.035
hsa-miR-20b-5p	*ESR1, BRCA1, CDKN1A, EFNB2, EPHB4*	20	−0.860	0.036
hsa-miR-126-3p	*SPPL2A, ZSWIM1, PIGS, ZNF257, ZNF543*	44	0.316	0.038
hsa-miR-101-5p	*PRKDC, ATM, EZH2, FOS, STMN1*	12	−1.359	0.040
hsa-miR-23b-3p	*PLAU, MET, CA2, RUNX2, CCNG1*	31	0.338	0.041
hsa-miR-942-5p	*CDKN1A, GSK3B, IFI27, NFKBIA, SFRP4*	6	−0.348	0.043
hsa-miR-451a	*MIF, ADAM10, AKT1, BCL2, CDKN2D*	22	−0.707	0.043
hsa-miR-339-3p	*FOXO1, MCL1, NFKB1*	3	0.483	0.044
hsa-miR-140-3p	*CD38, COL4A1, FN1, GPC1, ITGA6*	9	−0.389	0.044
hsa-miR-485-3p	*NTRK3, MAT1A, NFYB, PPARGC1A, SLC40A1*	6	−0.993	0.045
hsa-miR-324-5p	*GLI1, SMO, ETS1, SP1, MTFR1*	6	−0.860	0.047
hsa-let-7c-5p	*MYC, HMGA2, TGFBR1, BCL2L1, CASP3*	28	0.506	0.048
hsa-miR-155-5p	*AGTR1, RHOA, ETS1, MEIS1, FOXO3*	112	0.432	0.049

**Table 3:** **Gene ontology analysis.** Gene set enrichment analysis using terms within GO categories (biological process, cellular component, molecular function) was applied to extract biological meaning from the identified differentially expressed transcripts and predicted mRNA targets. The top 10 GO-significant categories associated with differentially expressed circulating miRNAs in patients with positive SLNs are shown. “Counts” refers to the ratio between the number of enriched differentially expressed miRNAs and the total number of miRNAs assigned to these terms.

GO ID	GO Term	Counts	q-Value
	**BIOLOGICAL PROCESS**		
0048732	Gland development	30/130	1.7 × 10^−18^
0048608	Reproductive structure development	30/130	1.7 × 10^−18^
0061458	Reproductive system development	30/130	1.7 × 10^−18^
1901652	Response to peptide	29/130	6.62 × 10^−16^
0050678	Regulation of epithelial cell proliferation	25/130	6.73 × 10^−15^
0048545	Response to steroid hormone	25/130	8.22 × 10^−15^
0070482	Response to oxygen levels	25/130	1.67 × 10^−14^
0050673	Epithelial cell proliferation	25/130	1.17 × 10^−13^
0071900	Regulation of protein serine/threonine kinase activity	26/130	1.72 × 10^−13^
0097305	Response to alcohol	19/130	1.1 × 10^−12^
0045785	Positive regulation of cell adhesion	23/130	1.16 × 10^−12^
	**CELLULAR COMPONENT**		
0005667	Transcription factor complex	19/131	1.14 × 10^−11^
0044798	Nuclear transcription factor complex	14/131	1.25 × 10^−10^
0090575	RNA polymerase II transcription factor complex	13/131	3.02 × 10^−10^
0000785	Chromatin	18/131	7.87 × 10^−07^
0045121	Membrane raft	14/131	1.51 × 10^−06^
0098857	Membrane microdomain	14/131	1.51 × 10^−06^
0098589	Membrane region	14/131	2 × 10^−06^
0000790	Nuclear chromatin	13/131	8.06 × 10^−06^
0044454	Nuclear chromosome part	15/131	2.58 × 10^−05^
1990909	WNT signalosome	4/131	2.58 × 10^−05^
	**MOLECULAR FUNCTION**		
0000978	RNA polymerase II proximal promoter sequence-specific DNA binding	22/130	4.96 × 10^−10^
0000987	Proximal promoter sequence-specific DNA binding	22/130	5.91 × 10^−10^
0019902	Phosphatase binding	15/130	5.91 x^−10^
0001228	DNA-binding transcription activator activity, RNA polymerase II-specific	19/130	1 × 10^−08^
0031625	Ubiquitin protein ligase binding	16/130	1.49 × 10^−08^
0019903	Protein phosphatase binding	12/130	1.87 × 10^−08^
0044389	Ubiquitin-like protein ligase binding	16/130	2.48 × 10^−08^
0003682	Chromatin binding	19/130	3.39 × 10^−08^
0001085	RNA polymerase II transcription factor binding	10/130	4.99 × 10^−07^
0033613	Activating transcription factor binding	8/130	8.48 × 10^−07^

## Data Availability

The original contributions presented in this study are publicly available at the Sequence Research Archive under ID PRJNA748920 and are available for download here: http://www.ncbi.nlm.nih.gov/bioproject/748920 (accessed on 22 July 2021).

## Supplementary Materials

The following supporting information can be downloaded at https://www.mdpi.com/article/10.3390/ijms241713293/s1.
