# Supplementary material for: Circulating miRNA Expression Is Inversely Correlated with Tumor Tissue or Sentinel Lymph Nodes in Estrogen Receptor-Positive Early Breast Cancer Patients
Source: Int J Mol Sci. 2023 Aug 27;24(17):13293. doi: 10.3390/ijms241713293 (PMC10487825; doi:10.3390/ijms241713293)
Supplement: Supplementary file 1 [file ijms-24-13293-s001.zip › ijms-2561617-supplementary.pdf]

Figure S1. RNA-sequencing quality check. (A) Read length distribution. (B) Summary of the number of ambiguous bases compared to all bases. The x-axis shows the percentage range and the y-axis the number of sequences featuring particular percentages normalized to the total number of sequences (C) Coverage of the number of sequences that support the individual base positions. The y-axis shows the number of sequences covering the respective base positions normalized to the total number of sequences. (D) Quality distribution per sequence. Summary of the distribution of average quality scores. The quality of a sequence is calculated as the arithmetic mean of its base qualities. The x-axis shows the PHRED range and the y-axis shows the number of sequences observed at a particular quality score normalized to the total number of sequences.

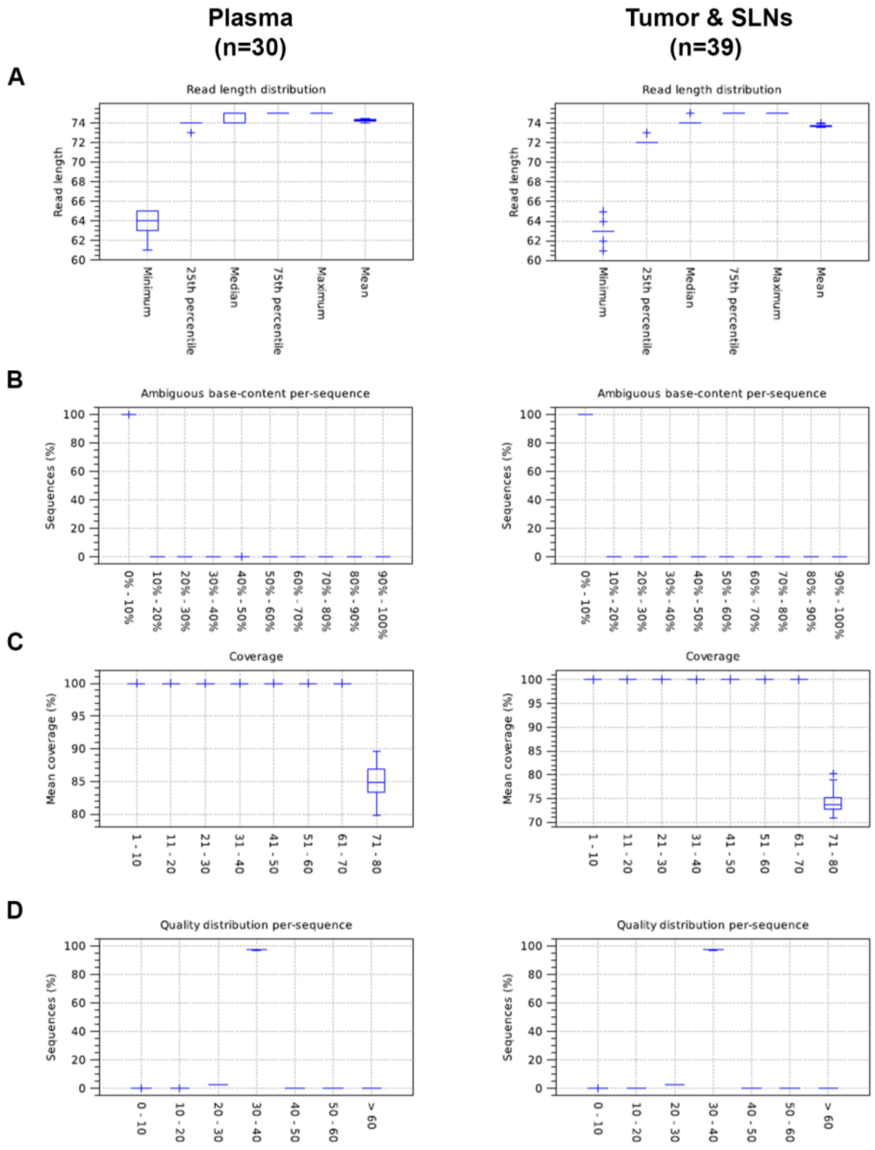

Figure S2. Summary of the mapping results. (A) Sequence length distribution after adapter trimming and filtering. The peak around 20-24 nucleotides corresponds to miRNAs. (B) Genome mapping results for plasma and tissue samples. The box plot shows the percentage of mapped reads. The y-axis shows a log2 scale.

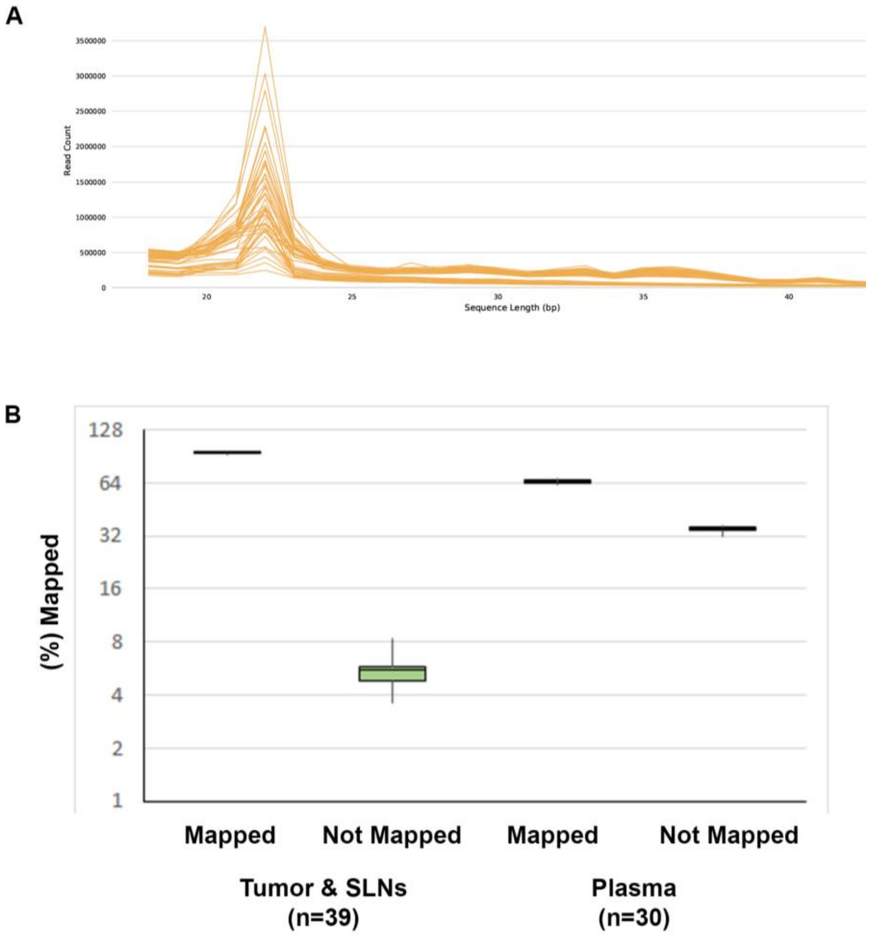

Figure S3. Unsupervised analysis and hierarchical cluster analysis. Heat map representing an unsupervised, hierarchical cluster analysis of (A) Plasma vs LN, (B) Plasma vs tumor tissue and (C) Tumor vs LN. Each row represents one miRNA and each column represents one sample. The color represents the relative expression level of a miRNA across all samples. The color scale shows the expression level above (purple) or below (green) the mean.

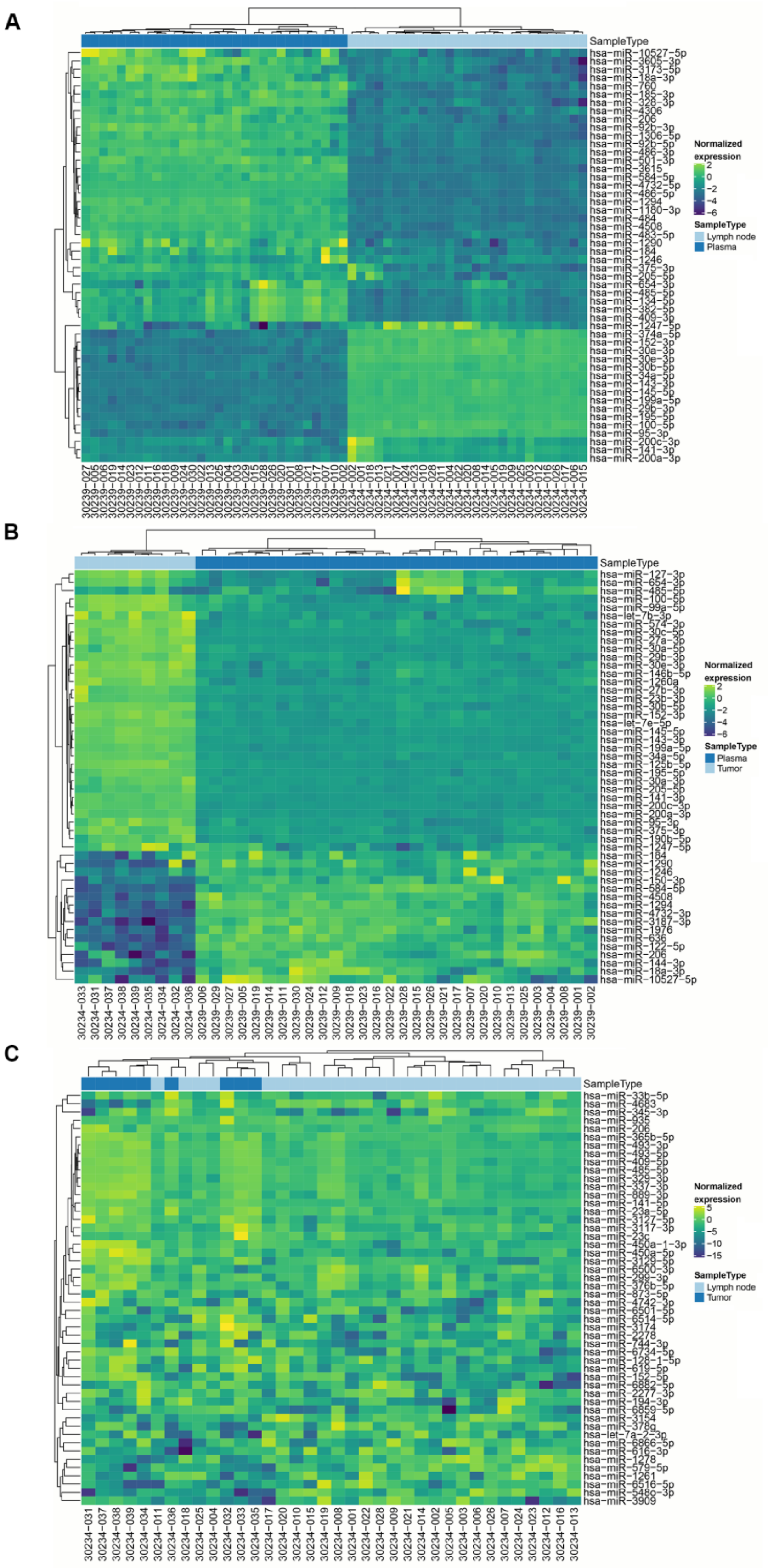
A
SampleType
Normalized expression
2
0
−2
−4
−6
SampleType
Lymph node
Plasma
B
SampleType
Normalized expression
2
0
−2
−4
−6
SampleType
Plasma
Tumor
C
SampleType
Normalized expression
5
0
−5
−10
−15
SampleType
Lymph node
Tumor

Figure S4. Spearman correlation analysis. Scatterplots show a sample-to-sample comparison between different tissues from the same patient. The scatterplots show the log expression of miRNAs expression and the Spearman correlation coefficient (r_s) for each comparison.

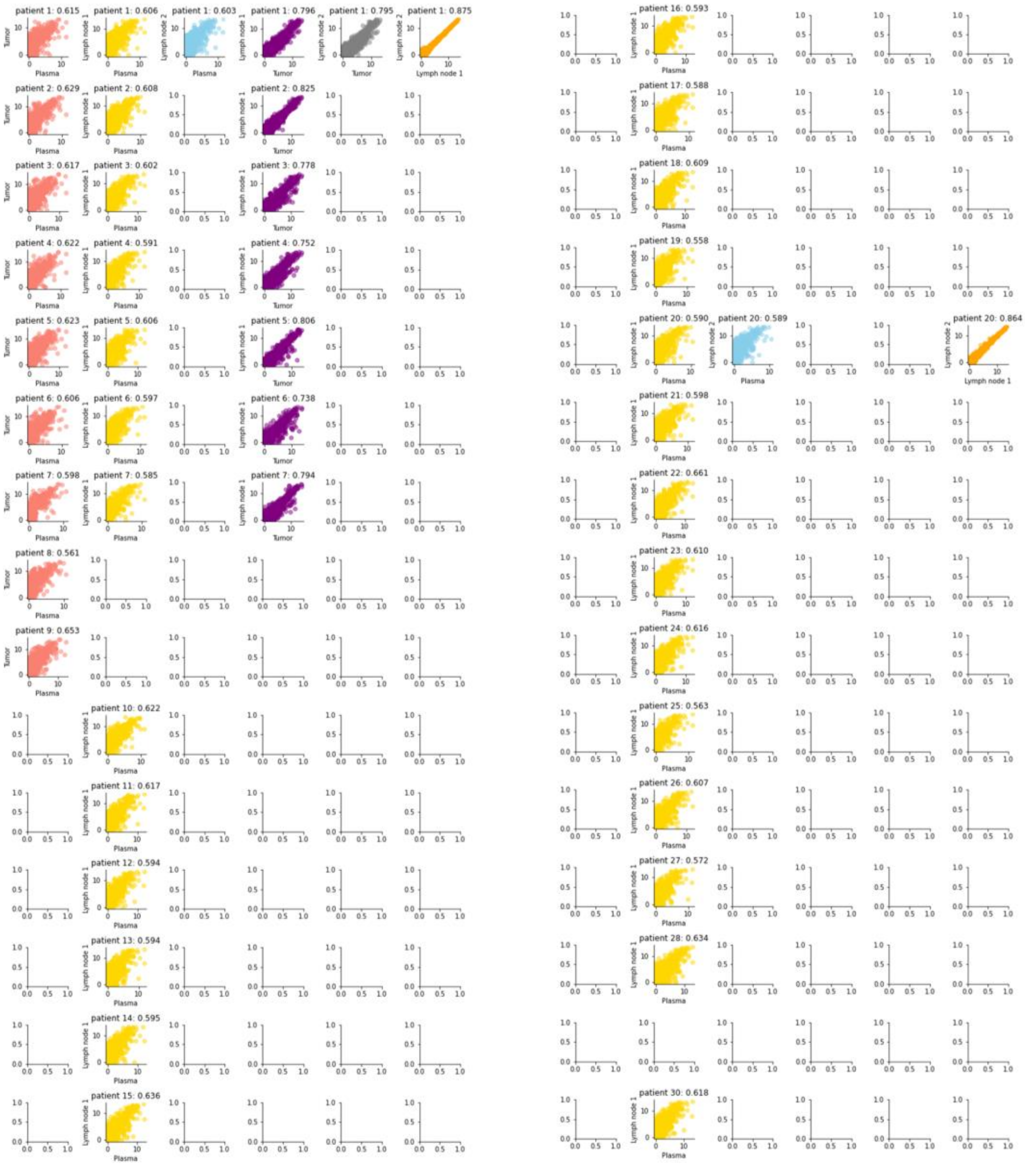

Table S1. Sample codification. A total of 30 patients were included in this study. Each row represents a patient and shows the availability for each sample, the RNA-seq codification number assigned and the SLN metastatic status. The CK19 copy number based on the OSNA assay is indicated in those patients with positive SLNs. P (plasma), L (sentinel lymph node), T (tumor tissue) CK19# (cytokeratin-19 copy number), n/a (not available).

Table S2. Normalized e**xpression of miRNAs**. MiRNAs were mapped and counted using the miRbase 22 database. Transcripts per million (TPM) was used as a normalization procedure to correct for differences in sequencing depth and to quantified each RNA species.

Table S3. Differentially expressed circulating miRNAs. Data shows the most significant differentially expressed miRNAs according to the metastatic status of patients.

Table S4. Validated target genes for each differentially expressed miRNA.

Table S5. Gene ontology analysis. The data shows a complete list of all the biological processes, cellular compartment and molecular functions GO terms associated with the differentially expressed circulating miRNAs according to the locoregional metastatic status of breast cancer patients.
